# Hydrogels in Burn Wound Management—A Review

**DOI:** 10.3390/gels8020122

**Published:** 2022-02-15

**Authors:** Agnieszka Surowiecka, Jerzy Strużyna, Aleksandra Winiarska, Tomasz Korzeniowski

**Affiliations:** 1 East Center of Burns Treatment and Reconstructive Surgery, 21-010 Leczna, Poland; jerzy.struzyna@gmail.com (J.S.); winiarskaaleksandra0706@gmail.com (A.W.); t.korzeniowski@gmail.com (T.K.); 2 Department of Plastic Surgery, Reconstructive Surgery and Burn Treatment, Medical University of Lublin, 21-093 Lublin, Poland; 3 Chair and Department of Didactics and Medical Simulation, Medical University of Lublin, 20-093 Lublin, Poland

**Keywords:** burns, hydrogels, dressing

## Abstract

Inert hydrogels are of a great importance in burn first aid. Hydrogel dressings may be an alternative to cooling burn wounds with streaming water, especially in cases of mass casualty events, lack of clean water, hypothermia, or large extent of burns. Hydrogels that contain mostly water evacuate the heat cumulating in the skin by evaporation. They not only cool the burn wound, but also reduce pain and protect the wound area from contamination and further injuries. Hydrogels are ideally used during the first hours after injury, but as they do not have antimicrobial properties *per se*, they might not prevent wound infection. The hydrogel matrix enables incorporating active substances into the dressing. The active forms may contain ammonium salts, nanocrystal silver, zinc, growth factor, cytokines, or cells, as well as natural agents, such as honey or herbs. Active dressings may have antimicrobial activity or stimulate wound healing. Numerous experiments on animal models proved their safety and efficiency. Hydrogels are a new dressing type that are still in development.

## 1. Introduction

**Figure 1:**
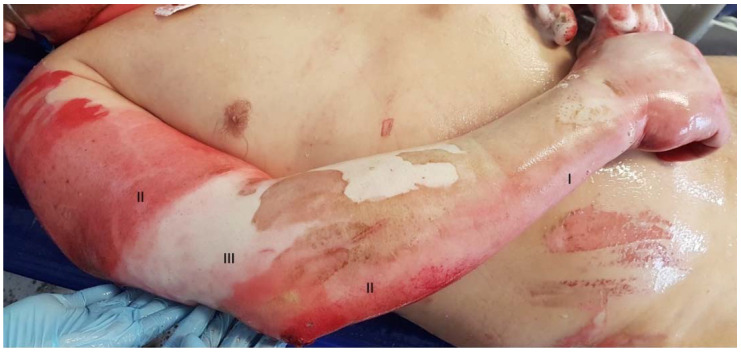
The depth of the burn wound evaluated at the admission to the burn unit. **I**—first degree/superficial; **II**—second degree, intermediate-depth; **III**—third degree/deep.

The skin serves as an anatomical barrier against pathogens and protects internal organs from damage from the external environment, maintains a balance between the system and the environment by controlling evaporation, and plays a role in thermoregulation. The definition of a burn wound [1] is an injury to the integrity of the skin and subcutaneous tissues caused by heat (flames, fluids, solid objects), extreme cold, electricity, chemicals, or radiation [2,3,4]. This injury initiates metabolic and molecular lesions that can lead to tissue necrosis and eschar formation [5]. In the central areas of the burn wound, the coagulation zone, capillary circulation is blocked, and death of cells is observed. The stasis zone surrounds the coagulation zone. It is an area of the skin with blood circulation impairment and risk of conversion to a full-thickness burn. Most peripherally, there is a zone of inflammation/hyperemia where pro-inflammatory factors induced in response to trauma cause erythema and oedema [6,7]. Superficial burns involve only the epidermis. Superficial partial-thickness burns extend into the superficial portion of the dermis. Deep burns involve a deeper layer of the reticular dermis, or the entire dermis (Figure 1).

Massive thermal damage to the skin leads to hypovolemic shock or multiorgan failure and increases risk of systemic infections. Proper wound management and systematic treatment prevent burn wound conversion and burn wound infection. There are many dressings available on the market that can be used in burns. Hydrogels in various forms are among the most frequently used types of dressings due to their specific features.

## 2. Burn Wound Cooling

**Figure 2:**
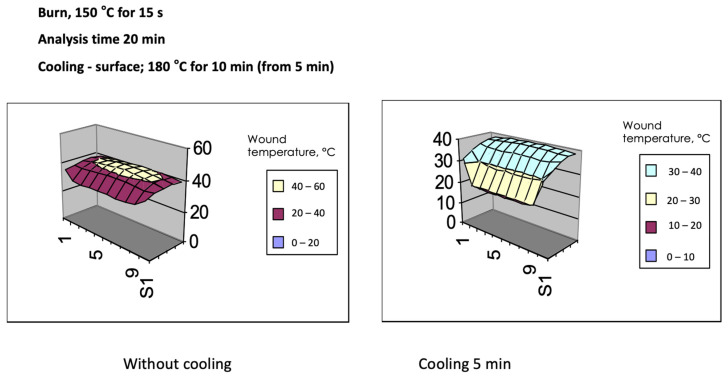
Analysis of the temperature of the burn wound (animal model). Thermal injury from heat at 150 °C for 15 s. (**left**) Change in the temperature of the burn wound without cooling, assessed 10 min after the burn; (**right**) decrease in the temperature of the injured tissues after 10 min of cooling commenced 5 min after burn.

The skin serves an anatomical barrier for pathogens, protects internal organs from damage, maintains a balance between the system and the environment, controls water, and plays role in thermoregulation. Immediately after the thermal injury occurs, the burn wound is only potential, as the disruption of tissues is not instantaneous. In thermal burns, heat energy is accumulated in the skin during the first 15 min and can be transferred to deeper layers or spread by means of different mechanisms, including convection, evaporation, or penetration. Directly after the burn occurs, the heat absorbed by the superficial layers of the skin is transferred deeper into the muscles and skin, as it is into the surrounding air. The skin consists primarily of water, which conducts heat 20 times faster than air. Burn wound cooling improves removal of heat from tissues and prevents deepening of the burn wound [8]. Burn wound cooling has been known since Galen and was popularized by Sorensen in the 1960s. Cooling by means of decreasing the temperature of the skin reduces the extent of the injury, protects the basal membranes, and reduces scarring [9,10]. Figure 2 presents the effect of burn wound cooling on burn wound temperature. The analysis of the temperature of the burn wound was performed by Strużyna et al. on an animal model. The thermal injury was caused by heat at 150 °C for 15 s. There are three factors that should be considered in terms of wound cooling: the temperature of cooling, the area of cooling, and the time of cooling. Ice and extreme cold can exacerbate tissue damage and increase the area of necrosis. According to the rule of 15, often used by emergency services, cooling with streaming water should be performed with water at 15 °C, from a distance of 15 cm and for a period of 15 min. During the first 15 min of cooling, the temperature of the wound stabilizes, there is an analgesic effect, and vascular spasm is irrelevant, especially in small burn wounds. Further cooling leads to hypothermia and does not affect the depth of the burn, but might be harmful instead [11]. However, it can also have an anti-inflammatory effect [12]. In the experimental models, it was proved that cooling a burn at 16 °C for 20 min was the most favorable within first hour after the burn [13,14]. Most burn associations recommend burn wound cooling with tap water, however, there are some concerns regarding the time of cooling. According to WHO recommendations, cooling a burn wound with tap water should be considered if fewer than 3 h have passed since the injury [15]. One of the main risks of cooling is hypothermia [16]. Streaming water, especially in ‘low and middle-income countries’, might not be the best cooling agent, and may be the source of wound contamination. What is more, in mass events, there might not be access to a source of streaming water [17].

Hydrogel dressings may be an alternative to cooling burn wounds with streaming water, especially in cases of mass casualty events, lack of clean water, hypothermia, or large extent of burns. Hydrogels in sheets that contain 96% water are used in first aid. They evacuate the heat cumulating in the skin by evaporation. The higher the water content, the better the wound cooling effect the dressing provides. Hydrogels are easy to use and available in different forms. Inert hydrogel sheets containing 96% water are included in the equipment of the US Marine Navy, and ambulances around the world. Hydrogels in masks are very useful in facial burns, whereas sheets can be also used for hand burns. The capacity of hydrogels to bind water molecules is due to their hydrophilic groups, such as –NH_2,_ –COOH, –OH, –CONH_2_, or –SO_3_H [18].

**Figure 3:**
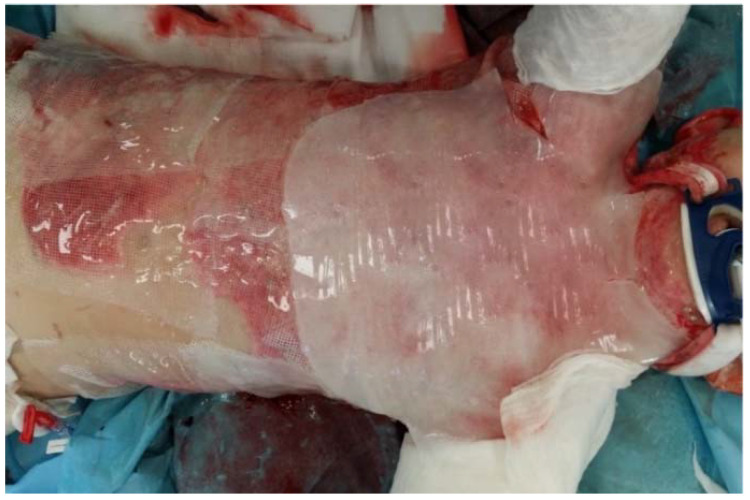
Application of hydrogels sheets in pediatric burns.

In burn management, hydrogels are of a great importance as first aid dressings [19,20]. They not only cool the burn wound, but also reduce pain and protect the wound area from contamination and further injuries [21]. These properties make hydrogels a great dressing for transportation and evacuation. During cooling with hydrogels, the obtained temperature is about 20.5 °C on the surface of the wound, whereas at a depth of 1–3 mm it is about 33 °C [14]. What is more, these products provide an analgesic effect and can be safely used as a first aid dressing even in pediatric patients [22] (Figure 3). The water content in hydrogels is important in cooling, as water stabilizes the temperature of the wound. Hydrogels in sheets can be safely used as a first aid dressing even in a pediatric group of patients. They reduce pain and cool the burn wound. The sheet can be applied even on large surfaces without the risk of hypothermia. 

## 3. Hydrogels in Burn Wound Management

First degree burns are superficial and involve only the epidermis. They heal spontaneously. Second-degree burns can be superficial or partial thickness and extend into the superficial portion of the dermis. They manifest as visible blisters filled with clear fluid, are moist, and very painful. When properly treated, they have a potential to heal spontaneously within 2–3 weeks. If the thermal damage involves a deeper layer of the dermis, reticular dermis and deeper, or the entire dermis, a third-degree burn or a full-thickness burn is recognized. Early excision of deep burns is recommended, as dead skin cells are a source of wound infection. Fourth-degree burns extend through the entire skin into fat, muscle, and bone and require surgical excision.

Hydrogels are defined as systems with at least two components, in which one component is a hydrophilic polymer, insoluble in water due to its chains being linked in a spatial network, whereas the other component is water. Hydrogels are a relatively new group of dressing materials. The first hydrogel, which was intended to be used for the production of contact lenses, was developed in 1960 by Wichterle and Lim. Hydrogels are polymers containing up to 96% water [23]. Such high water content affects the properties of the dressings—they ensure adequate wound hydration, strongly absorb the exudate, and induce autolysis of the devitalized tissues [23]. They are very pliable and soft, which makes them atraumatic in use [23]. Importantly, they are immunologically neutral [24]. Hydrogels are available in several forms, including solid sheets or semi-liquid gels [25]. 

Owing to the use of radiation technology, hydrogel dressings containing as much as 96% water forming a stable and mechanically strong hydrogel patch, with a thickness of approximately 3 mm. Such high water content (the sheet of the dressing itself is not wet) affects the properties of the dressings—they ensure adequate wound hydration, strongly absorb the exudate, and induce autolysis of the devitalized tissues. They are very pliable, soft, and immunologically inert. Hydrogels accelerate wound healing in various phases, accelerate autolytic wound cleansing, and reduce pain [26]. Water absorption is the most important feature of hydrogels and results from on the cross-linking of the dressing’s structure and presence of hydrophilic or hydrophobic monomers [27]. The features of hydrogels are also dependent on the crosslinking substance, such as N,N′-methylene-bis-acrylamide or 1,1,1- trimethylolpropane trimethacrylate (and ethylene glycol dimethacrylate or poly(ethylene glycol) diacrylate (PEG)) [27]. Their degradation is processed by hydrolysis [26]. Hydrogel transparency enables constant evaluation of the process of healing [26]. Hydrogels absorb and retain wound exudate, as well as promoting fibroblasts and epithelium migration [26,28]. They also stimulate the process of autolysis and wound bed autodebridement [28]. Injectable hydrogels have an on-demand resorption capacity and can be easily applied, as the product fills the wound bed and can be dissolved with amino acids [29]. An injectable hybrid hydrogel crosslinked with iodine-modified 2,5-dihydro-2,5-dimethoxy-furan and chitosan, improved adhesion, migration, and proliferation of human keratinocytes and fibroblasts as well as improved neovascularization [30].

Hydrogels accelerate the healing of burn wounds (superficial and moderate-thickness burns) compared with standard procedures, such as paraffin dressings [25,31]. What is more, changing hydrogel dressings caused less pain and the need for dressing changes was reduced [25]. Similar results were achieved for hydrogel fibers. The main drawback of hydrogel fibers is their high cost. However, they protect against thermal damage, reduce pain, and eliminate unpleasant smells [32]. Reduction of pH and erythema by transparent hydrogels allows for better assessment of even minor changes, which can otherwise be difficult when edema is present [33]. 

## 4. Types of Hydrogels

**Figure 4:**
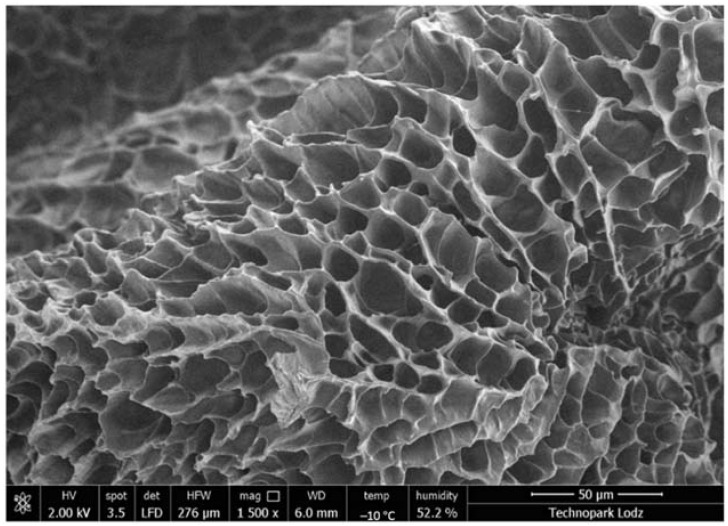
A microporous structure of the inert hydrogel in which different active substances can be incorporated. Photo courtesy of KikGEL Poland, Lodz.

Hydrogels are rapidly developing dressings. Their microporous structure makes them an attractive vehicle for active substances (Figure 4).

**Figure 5:**
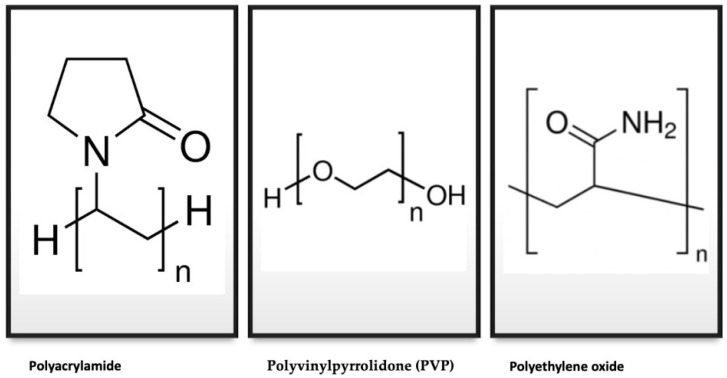
Chemical structures of the most commonly used synthetized hydrogels.

Hydrogel dressings can be divided, according to the type of polymer used, into natural and synthetic ones or, depending on the substances added, to inert and active [34]. Natural hydrogels are based on chitosan, cellulose, alginate, dextran, or hyaluronic acid [35]. Hydrogels can be synthetized from polymeric chains of polyacrylamide, polyethylene oxide, or polyvinylpyrrolidone (PVP) [36]. The chemical structure of the polymers is shown in Figure 5.

Hydrogels are available in various sizes, shapes, and forms (sheets or amorphic/gel) [37]. Adding active substances to hydrogels, however, decreases the water content. Tested on animal models, available dressings showed low irritation index, low adverse event rates, and accelerated wound healing at different stages of burn wound healing [38]. 

Stoica et al. divided hydrogels into inert and active hydrogels [34]. The active forms may contain ammonium salts [39], nanocrystal silver, zinc, growth factor, cytokines, or cells, as well as natural agents, such as honey or herbs [34]. Table 1 summarizes active hydrogels with various substances that can potentially be used in a burn wound treatment. The connections in the structure of the hydrogel enables incorporating various active substances in the matrix. Combined hydrogels differ from inert hydrogels, and water acts as a vector for active substances. The features of active hydrogels also differ from those of inert water hydrogels.

## 5. Antimicrobial Activity

The skin, the largest organ in the body, provides primary protection against a wide variety of pathogens, by acting as a physical barrier. If it is damaged, bacteria can directly infiltrate the body, resulting in infection. Infection is the most common cause of mortality in burn patients. Management of burn wound infection involves the use of topical antimicrobial agents, systemic antibiotics, early debridement of dead tissue, and the use of appropriate dressings. The latter should be considered an essential infection control tool, as many are capable of physically preventing the transmission of pathogens [56,57,58,59].

Hydrogels are ideally used during the first hours after injury, but as they do not have antimicrobial properties *per se*, they might not prevent wound infection. The connections in the structure of the hydrogel prevent bacteria from reaching the wound surface, while enabling water evaporation and oxygen penetration to the wound [60,61]. Most the hydrogel sheets do not have an antimicrobial agent incorporated. To address infections, betadine of chlorhexidine dressings can be applied over the hydrogel [36], or the hydrogel structure can be enriched with active substances. The structure of hydrogels can act as a vector for active antimicrobial substances. Ionized silver nanoparticles incorporated in hydrogel structures are used to create even more effective dressings and unfavorable conditions for bacterial growth [40,62,63]. Studies showed that dressings with this technology effectively inhibit the growth of pathogens, such as *Pseudomonas aeruginosa*, *Staphylococcus aureus* (MSSA and MRSA), and *Enterococcus faecalis* (VRE), and they also delay the formation of biofilm on the wound surface [41,62]. Hydrogels containing silver nanoparticles are not cytotoxic, and as much as 82% of the silver contained in the dressings is released during the first 72 h after application [40,42]. However, a 2010 study by Grippaudo showed that hydrogel dressings did not reduce the incidence of generalized *Pseudomonas aeruginosa* infections requiring intravenous antibiotics [25,41,64]. A hydrogel with oxidized dextran (ODex), adipic dihydrazide grafted hyaluronic acid (HA-ADH), chitosan (HACC), and silver nanoparticles potentially prevent *E. coli*, *Staphylococcus aureus* and *Pseudomonas aeruginosa* infections [65]. Hydrogels also have antifungal properties [41]. Microporous chitosan hydrogen/nano zinc oxide composite bandages showed good antimicrobial activity due to release of reactive oxygen species (ROS) by zinc [43]. Hydrogels containing 1% sulfadiazine improved wound healing [44]. Honey can be incorporated in the hydrogel structure, reducing secretion of proinflammatory cytokines (L-1α, IL-1β, and IL-6) [45,46]. Colistin can also be incorporated in the hydrogel matrix. Such a combination was shown to be effective against *Pseudomonas aeruginosa* [45]. Furthermore, minocycline and gentamicin can be incorporated in the hydrogel matrix [47]. The main drawback of the dressing might be quick release of the antibiotic.

Porous polysaccharide-based hydrogels can be cross-linked with active substances, such as oils extracted from aromatic plants, including terpenoids and terpenes. Essential oils are active against Gram-positive and Gram-negative bacteria and have antioxidative capacities [48].

## 6. Promotion of Wound Healing

Hydrogels containing chitosan promote fibroblast proliferation and secretion of collagen type III, as well as macrophage migration and burn wound autolysis [38]. Hydrogels can improve neovascularization. Cells from the wound can infiltrate the structure of the hydrogel even up to 100 µm within the first 24 h [43]. In histopathological specimens, new vessels were observed, and the dressing accelerated epithelial proliferation [49]. Dextran hydrogels stimulate neovascularization by increasing detectable VEGFR2 and stimulating luminal structure formation [50,66]. Hydrogels can also be a vector for plasmid transfer. Plasmid DNA encoded with vascular endothelial growth factor (pDNA-VEGF) and anti-inflammatory resveratrol were incorporated into a hydrogel scaffold and tested on a rat model. The density of CD31 and α-SMA, characteristic of new vessels, was increased. What is more, the levels of IL-1β and TNF-α in the treated wounds were similar to levels observed in a healing wound, with upregulation within 7 days and downregulation in the late stage of wound healing, unlike in the case of untreated wounds, in which presence of proinflammatory pathways was observed for longer [51].

Adipose derived stem cells can also be incorporated in PEG hydrogel [52]. Such dressings were used in full-thickness thermal burns in a rat model, and improved wound closure (95% vs. 79% with a saline gauze). They stimulated granulation and remodeling of the dermal layer. Adipose derived stem cells (ADSC) are multipotent cells that do not proliferate in vivo but act as regulatory cells [67]. They are characterized by expression of CD34+, CD44+, CD31−, and CD45− on the surface of cell membranes [68]. They have a high ability to stimulate regeneration and promote neoangiogenesis in the dermis [68,69,70]. They secrete many growth factors, included for fibroblasts (FGF), the endothelium (VEGF), as well as anti-inflammatory cytokines. ADSCs stimulate tissue regeneration, promoting the secretion of proteins and glycosaminoglycans of extracellular matrices, such as collagens I, II, III, and V, elastin, but also metalloproteinases [71,72]. ADSCs activate skin fibroblasts through various pathways, including Wnt/β-catenin, PI3K/Akt, through insulin-like growth factor (IGF), and IL-1 [71]. ADSCs also have paracrine properties. They secrete exosomes and microbubbles containing proteins, nucleic acids, lipids, and enzymes [72]. The mechanisms triggered by ADSC are non-specific pathways of the immune response [67].

Low levels of reactive oxygen species (ROS) promote wound healing by stimulating cell migration and angiogenesis but excessive ROS can lead to cellular damage and impair healing processes. Therefore, maintaining the balance of redox in cells is also beneficial in the treatment of a burn wound. In order to improve wound healing, hydrogel dressings with antioxidant functions have appeared, creating more favorable conditions for the wound healing. These types of dressings can remove excess ROS from burn wounds to reduce oxidative stress and ultimately achieve enhanced wound repair [53,73,74,75,76].

Extra cellular matrix hydrogels promote natural healing capacities and may contain hyaluronic acid and gelatin [77]. Microcapillary gelatin-alginate hydrogel with infused anti-TNF α decreased the rates of burn wound conversion to full-thickness in a murine model [54]. Alginate-containing hydrogels present hemostatic properties as well [78]. Furthermore, growth factors can be incorporated in the hydrogel matrix. A collagen hydrogel with incorporated bFGF and silver sulfadiazine increased wound re-epithelialization. bFGF stimulated migration of fibroblasts and synthesis of collagen, as well as promoted angiogenesis. It also promoted wound healing by pathways for nerve growth factor (NGF), tropomyosin-receptor kinase A (TrkA), p-TrkA, extracellular regulated kinase 1 and 2 (ERK1/2), p-ERK1/2, NF-kβ, and p-NF-kβ [55].

## 7. Thermo-Sensitive Hydrogel

Physical properties of resembling living tissues provide unique properties of hydrogels. Traditional inert hydrogels cool the burn wound. Thermo-sensitive hydrogels are an excellent example of smart hydrogels and are the best-studied polymer systems. Thermogels’ gels can be divided into negatively thermo-sensitive and positively thermo-sensitive hydrogels [79]. Temperature changes can induce solution to gel transition in the case of thermo-responsive hydrogels. Thermo-responsive hydrogels can undergo phase transition or swell/deswell as ambient temperature changes, endow the drug delivery system with enhanced local drug penetration, desirable spatial and temporal control, and improved drug bioavailability. The most commonly used thermogels are poly(N-isoprolylacrylamide), pNiPAAm 32, poly(ethylene glycol), PEG 120, poly(propylene glycol), PPG 50, poly(methacrylic acid), PMAA 75, poly(vinyl alcohol), PVA 125, poly(vinyl pyrrolidone), PVP 160, and methylcellulose, MC [80]. The phase transition at physiological temperatures, in addition to injectability and easy drug loading in sol phase, enables thermogels to be widely used in drug delivery systems [37]. Hydrogel properties led to the development of systems with controlled drug release, high drug concentration in the tumor site, prolonged topical residence time, sustained drug release, and reduced systemic side effects because of minimal invasiveness. To date, thermogel delivery systems were used in transdermal [81,82,83], ocular [84,85,86], nasal [87,88,89], buccal [90,91] drug delivery systems, as well as anti-tumor [92,93,94] drug delivery systems in cancer and chronic diseases treatment. 

In recent years thermogels were tested in three-dimensional cell/stem cell culture in order to regenerate tissues such as cartilage [95,96,97], bone [98,99] or nerves [100], and burn wounds [79,101]. Hydrogels with advanced properties have the ability to mimic the structure and biological properties of the native ECM [102], thus their use is tissue engineering is a promising direction of future research.

## 8. Clinical Application

**Figure 6:**
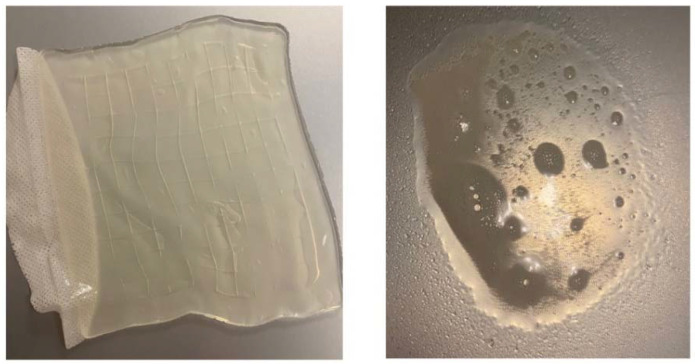
Types of hydrogels. On the **left**, an inert hydrogel in a sheet. On the **right** a semi-liquid, amorphic form.

Hydrogels are widely used in tissue engineering and clinical practice for a wide range of applications, including burn wound care. Hydrogels can be applied on a burn wound in the form of a solid sheets or semi-liquid gels (amorphic) (Figure 6).

**Figure 7:**
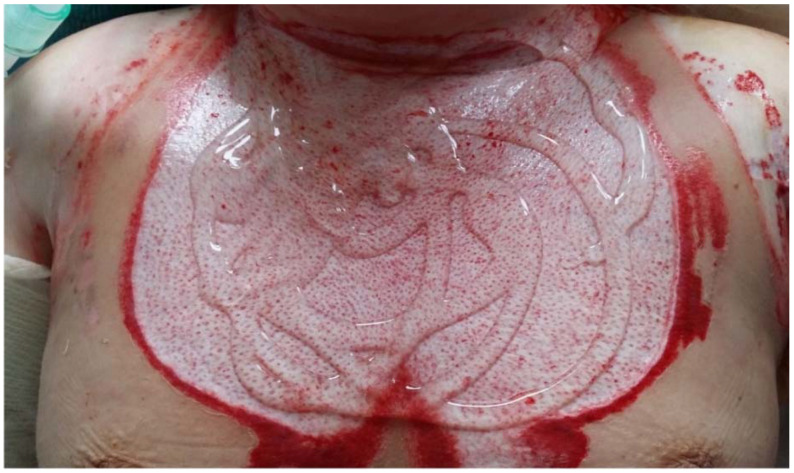
Application of amorphic hydrogels containing polyhexanide (PHMB) after enzymatic debridement.

Amorphic hydrogels containing polyhexanide (PHMB) can be used in burn wound treatment. A randomized controlled single-center study revealed that such hydrogel was superior to sulfadiazine ointment in terms of pain management and wound staining [103]. PHMB is recommended as an antiseptic in burn wound management [104]. A hydrogel scaffold with incorporated PHMB reduced the incidence of wound infection [105]. An amorphic hydrogel with PHMB can also be applied to stimulate ‘wet’ healing after enzymatic debridement with bromelain [106]. The hydrogel is applied in the third step of the procedure, after a wet-to-dry dressing post the procedure. It is usually applied over a meshed paraffin gauze. Enzymatic debridement of burn wounds using bromelain is becoming an increasingly appreciated method. Bromelain is derived from pineapple stems and indicated for the removal of dead tissue in thermal burns. This solution does not work on dry wounds, which is why a moist environment needs to be ensured. Hydrogels effectively maintain moisture balance in the wound and can therefore be used at several stages of the enzymatic debridement procedure. Longer soaking or specialist dressings, such as hydrogels, need to be applied to improve wound status before the procedure is carried out. The key aspect of post-debridement wound care involves preventing the wound from desiccation. If the wound dries, pseudoeschar will occur, which may necessitate additional surgical procedures and may also result in longer treatment and worse outcomes. Hydrogels are very effective and useful in maintaining wound moisture after the procedure is completed [107,108]; Figure 7 shows an example of use of a semi-liquid hydrogel to maintain adequate moisturization of wound bed after enzymatic debridement. 

Hydrogels can also be applied in facial burns. Burd et al. reported application of inert hydrogels later during the first day after the injury and on different types of burn wounds. Non-exudative partial-thickness facial burns cured under a hydrogel mask within 10 days, whereas exudative partial-thickness burns might require additional intervention [36]. Using a hydrogel mask in second-degree facial burns accelerates healing and reduces scarring [109]. In full-thickness burns, hydrogels can be applied on a meshed skin-graft using a ‘sandwich technique’ to improve graft adherence and healing. Hydrogel sheets can also be used on a donor site after skin is harvested for transplantation. The dressing can be removed even after 10 days, when it becomes ‘crispy’. The donor site will epithelialize under the hydrogel [36]. Table 2 summarizes clinical studies regarding hydrogels.

Hydrogels are transparent and an ultrasound examination is feasible through the dressing [110]; however, different types of hydrogels can reduce transmissivity [111].

## 9. Conclusions

Hydrogels are safe and efficient in burn wound management. They can be used during all stages of burn wound treatment. Hydrogels are a dynamically developing group of dressings. Their structure makes them a good vehicle for active substances, including antimicrobials, wound healing promoting factors, biological agents, or growth factors. Most of the available hydrogel dressings have been tested on animal models. Multicenter clinical observations need to be performed to evaluate the actual clinical efficacy.

## Figures and Tables

**Table 1:** Possible hydrogel modifications and potential use in burn wound treatment.

Study	Study Type	Dressing Activity	Patients and Methods	Outcomes
Structural effects in photopolymerized sodium AMPS hydrogels crosslinked with poly(ethylene glycol) diacrylate for use as burn dressings [27]	Experimental	Wound healing	Hydrogel sheets were exposed to water binding, swelling and tested for cytotoxicity.	A potential for biomedical use as dressings for partial thickness burn
On-Demand Dissolvable Self-Healing Hydrogel Based on Carboxymethyl Chitosan and Cellulose Nanocrystal for Deep Partial Thickness Burn Wound Healing [29]	Experimental	Wound healing	An injectable hydrogel with carboxymethyl chitosan rigid rod-like dialdehyde-modified cellulose nanocrystal was administrated in a rat model after burn wound surgical debridement.	The hydrogel stimulates cell growth, is resoluble with amino acids and adjusts to the wound bed.
Dual Functionalized Injectable Hybrid Extracellular Matrix Hydrogel for Burn Wounds [30]	Experimental	Wound healing	An injectable hybrid decellularized crosslinked hydrogel derived from rat dermal tissue was tested for safety and efficiency.	The hydrogel containing cytokine and growth factors and was shown to be non-immunogenic and nontoxic.
Evaluation of healing activity of PVA/chitosan hydrogels on deep second degree burn: pharmacological and toxicological tests [38]	Experimental	Wound healing	Hydrogel containing chitosan was tested in a rat burn wound model. Toxicological test, irritation tests and histopathological analyses were performed.	Hydrogels containing chitosan accelerated wound healing at different times of the process. Had low irritation index.
Antimicrobial efficacy of a novel silver hydrogel dressing compared to two common silver burn wound dressings: Acticoat and PolyMem Silver [40].	Experimental	Antimicrobial activity	Hydrogel containing 2-acrylamido-2-methylpropane sulfonic acid sodium salt with silver nanoparticles was tested for antimicrobial activity	Silver containing hydrogels inhibited growth of MSSA, *Pseudomonas aeruginosa*, but did not decrease VRE. Nanocrystal dressing based on polyethylene showed superior antimicrobial properties.
Biocompatibility evaluation of a new hydrogel dressing based on polyvinylpyrrolidone/polyethylene glycol [41]	Experimental	Wound healing and antimicrobial activity	Hydrogel samples (PEG, PVP, agar and water) were evaluated for fibroblast cytotoxicity, antifungal and antibacterial properties.	The material was nontoxic, showed good antibacterial and antifungal actions against *Staphylococcus aureus, Staphylococcus epidermidis*, *Escherichia Coli* k12 but no effect on *Pseudomonas aeruginosa*.
Mechanical properties and in vitro characterization of polyvinyl alcohol-nano-silver hydrogel wound dressings [42].	Experimental	Antimicrobial activity	PVA-Ag hydrogels were examined for cytotoxicity, antibacterial features, swelling and drug delivery	PVA-Ag were not toxic for human fibroblast, and could be used in burn wound management.
Flexible and microporous chitosan hydrogel/nano ZnO composite bandages for wound dressing: in vitro and in vivo evaluation [43]	Experimental	Antimicrobial activity	Microporous chitosan hydrogen/nano zinc oxide composite bandages were evaluated for cell cytotoxicity, swelling, and antibacterial properties. Murine model of a burn wound	Dressing is not cytotoxic, improves wound healing and neovascularization.
Development and in vivo evaluation of silver sulfadiazine loaded hydrogel consisting polyvinyl alcohol and chitosan for severe burns [44]	Experimental	Wound healing	Murine model of a burn wound. Hydrogel containing 1% sulfadiazine tested for cytotoxicity and healing properties.	The dressing is safe and improved burn wound healing.
Gelam (Melaleuca spp.) honey- based hydrogel as burn wound dressing [45]	Experimental	Wound healing	A hydrogel with honey incorporated was tested on a rat model	Acceleration of wound healing and epithelialization was observed
Research on a novel poly (vinyl alcohol)/lysine/vanillin wound dressing: Biocompatibility, bioactivity and antimicrobial activity [46]	Experimental	Wound healing, antimicrobial activity	PVA hydrogels tested for antibacterial features and environmental scanning electron microscope (ESEM) and rat model	The dressing had antimicrobial activity and stimulated vessel formation and epithelization
Antibiotic-Containing Agarose Hydrogel for Wound and Burn Care [47]	Experimental	Antimicrobial activity	An agarose hydrogel with minocycline was tested for safety and antimicrobial features on a pig model.	The hydrogel showed 80% bioactivity after 7 days with much of the drug release within first 25 h.
Antibacterial polysaccharide-based hydrogel dressing containing plant essential oil for burn wound healing [48]	Experimental	Antimicrobial	Polysaccharide- based hydrogel with incorporated essential oils (eucalyptus, ginger and cumin) were examined regarding safety and antimicrobial activity.	Adding essential oils to hydrogels improved its antimicrobial activity against *Staphylococcus aureus* and *Escherichia Coli.* No indirect nor direct cytotoxicity was observed.
A Hydrogel-Based Localized Release of Colistin for Antimicrobial Treatment of Burn Wound Infection [49]	Experimental	Antimicrobial activity	A glycol chitosan/DF-PEG hydrogel loaded with colistin was tested for its safety and antimicrobial action.	Colistin was effectively released from the hydrogel and acted against *Pseudomonas aeruginosa*.
Dextran hydrogel scaffolds enhance angiogenic responses and promote complete skin regeneration during burn wound healing [50]	Experimental	Wound healing	Murine burn model applicated after early burn wound excision	The dressing improved neovascularization and wound healing
In situ formed anti-inflammatory hydrogel loading plasmid DNA encoding VEGF for burn wound healing [51]	Experimental	Wound healing	Hydrogel from chemically modified hyaluronic acid (HA), dextran (Dex), and β-cyclodextrin (β-CD) integrating resveratrol (Res) and vascular endothelial growth factor (VEGF) was tested on a rat model.	The novel dressing was proved to be safe and to improve wound healing. The density of CD31 and α-SMA, characteristic for new vessels, were increased. Levels of IL-1β and TNF-α in the treated wounds were similar to correctly healing wound.
Bilayer hydrogel with autologous stem cells derived from debrided human burn skin for improved skin regeneration [52]	Experimental	Wound healing	Human ADSCs incorporated in PEG hydrogel and applicated on full thickness burn wound rat model	ADSC/PEG hydrogels improved healing even of full thickness wounds and stimulated dermis remodeling
Non-stick hemostasis hydrogels as dressings with bacterial barrier activity for cutaneous wound healing [53]	Experimental	Antimicrobial activity, wound healing	A rabbit model was used to evaluate features of potentially hemostatic multifunctional hydrogel composed of poly (vinyl alcohol), human-like collagen (HLC) and sodium alginate (SA)	The hydrogels showed hemostasis, anti-protein absorption, and bacterial barrier activity. No cytotoxicity was observed.
Successful prevention of secondary burn progression using infliximab hydrogel: A murine model [54]	Experimental	Wound healing	Microcapillary gelatin- alginate hydrogel with infused anti-TNF α was tested for efficiency and safety in a murine model.	The novel dressing reduced depth of thermal injury and promoted wound healing by downregulation of proinflammatory cytokines.
bFGF and collagen matrix hydrogel attenuates burn wound inflammation through activation of ERK and TRK pathway [55]	Experimental	Wound healing	A collagen hydrogel with incorporated bFGF and silver sulfadiazine was tested in a rat model and evaluated for efficiency and safety.	The hydrogel promoted wound healing by NGF, stimulation of fibroblast proliferation, increasing neoangiogenesis. No serious cytotoxicity was observed.

**Table 2:** Clinical evidence for hydrogel application.

Study	Study Type	Patients and Methods	Outcomes
Pre-hospital management of burns by the UK fire service [20]	A questionary	62 UK fire and rescue services were questioned about first aid in burns	76% use hydrogel dressing, while 37% would cool the wound with hydrogel
Effectiveness of a hydrogel dressing as an analgesic adjunct to first aid for the treatment of acute pediatric burn injuries: a prospective randomized controlled trial [22]	A prospective randomised controlled trial	Children were enrolled into two groups: intervention with inert hydrogel or control with polyvinylchloride film	No significant between-group differences in pain scores were found between 17 paediatric burn patients who received hydrogel dressings and those who received standard care
Evaluating the use of hydrogel sheet dressings in comprehensive burn wound care [36]	A prospective clinical observation	50 burn wounds in 30 patients treated with hydrogel sheets. Full-thickness and partial-thickness burn wounds, as well as the donor areas were treated.	No adverse events were reported. The hydrogel dressing reduced pain, improved wound healing
Clinical safety and efficacy of a novel thermoreversible polyhexanide-preserved wound covering gel [104]	A randomized controlled single-center study	44 patients, test group—hydrogel with polyhexanide, control group—ointment with sulfadiazine	There was less pain and wound staining in the test group. Hydrogels were safe and effective.
Clinical Performance of Hydrogel-based Dressing in Facial Burn Wounds: A Retrospective Observational Study [109]	A retrospective observational study	21 patients with burn enrolled in the study, a hydrogel mask was used. Full epithelialization took 10.86 days	Hydrogel mask improved healing and reduced scarring in a group of patients with second-degree facial burns.

## Data Availability

Not applicable.

## References

[1] Herman T.F., Bordoni B. (2021). Wound Classification. StatPearls.

[2] Oltulu P., Ince B., Kokbudak N., Findik S., Kilinc F. (2018). Measurement of epidermis, dermis, and total skin thicknesses from six different body regions with a new ethical histometric technique. Turk. J. Plast. Surg..

[3] Chopra K., Calva D., Sosin M.D., Tadisina K.K., Banda A., De La Cruz C., Chaudhry M.R., Legesse T., Drachenberg C.B., Manson P.N. (2015). A comprehensive examination of topographic thickness of skin in the human face. Aesthetic Surg. J..

[4] Levy V., Lindon C., Zheng Y., Harfe B.D., Morgan B.A. (2007). Epidermal stem cells arise from the hair follicle after wounding. FASEB J..

[5] Demling R.H. (1985). Burn injury. Acute Care.

[6] Jackson D.M. (1953). The diagnosis of the depth of burning. Br. J. Surg..

[7] Jackson D.M. (1969). Second thoughts on the burn wound. J. Trauma Inj. Infect. Crit. Care.

[8] Demling R.H., Mazess R.B., Wolberg W. (1979). The effect of immediate and delayed cold immersion on burn edema formation and resorption. J. Trauma Inj. Infect. Crit. Care.

[9] Raine T.J., London M.D., Robson M.C., Heggers J.P. (1982). Progression of thermal injury: A morphologic study. Plast. Reconstr. Surg..

[10] De Camara D.L., Raine T., Robson M.C. (1981). Ultrastructural aspects of cooled thermal injury. J. Trauma Inj. Infect. Crit. Care.

[11] Wood F.M., Phillips M., Jovic T., Cassidy J.T., Cameron P., Edgar D.W. (2016). Steering Committee of the Burn Registry of Australia and New Zealand (BRANZ). Water First Aid Is Beneficial In Humans Post-Burn: Evidence from a Bi-National Cohort Study. PLoS ONE.

[12] Ashman H. (2018). Cooling of thermal burn injuries: A literature review. J. Paramed. Pract..

[13] Wright E.H., Tyler M., Vojnovic B., Pleat J., Harris A., Furniss D. (2019). Human model of burn injury that quantifies the benefit of cooling as a first aid measure. Br. J. Surg..

[14] Cuttle L., Kempf M., Liu P.-Y., Kravchuk O., Kimble R.M. (2010). The optimal duration and delay of first aid treatment for deep partial thickness burn injuries. Burns.

[15] Hughes A., Almeland S.K., Leclerc T., Ogura T., Hayashi M., Mills J.-A., Norton I., Potokar T. (2021). Recommendations for burns care in mass casualty incidents: WHO Emergency Medical Teams Technical Working Group on Burns (WHO TWGB) 2017–2020. Burns.

[16] Coats T.J., Edwards C., Newton R., Staun E. (2002). The effect of gel burn dressings on skin temperature. Emerg. Med. J..

[17] Strużyna J., Surowiecka A., Korzeniowski T. (2021). Letter to the Editor on Recommendations for burns care in mass casualty incidents: WHO Emergency Medical Teams Technical Working Group on Burns (WHO TWGB) 2017–2020. Burns.

[18] Wichterle O., Lím D. (1960). Hydrophilic Gels for Biological Use. Nature.

[19] Goodwin N.S. (2016). European Resuscitation Council 2015 burn 1st Aid recommendations–concerns and issues for first responders. Burns.

[20] Walker A., Baumber R., Robson B. (2005). Pre-hospital management of burns by the UK fire service. Emerg. Med. J..

[21] Hhauflaire-Uhoda E., Paquet P., Pierard G.E. (2008). Dew point effect of cooled hydrogel pads on human stratum conreum biosurface. Dermatology.

[22] Holbert M.D., Kimble R.M., Chatfield M., Griffin B.R. (2021). Effectiveness of a hydrogel dressing as an analgesic adjunct to first aid for the treatment of acute paediatric burn injuries: A prospective randomised controlled trial. BMJ Open.

[23] Daunton C., Kothari S., Smith L., Steele D. (2012). A history of materials and practices for wound management. Wound Pract. Res..

[24] Murphy S., Skardal A., Atala A. (2013). Evaluation of hydrogels for bio-printing applications. Biomed. Mater. Res..

[25] Wasiak J., Cleland H., Campbell F., Spinks A. (2013). Dressings for superficial and partial thickness burns. Cochrane Database Syst Rev..

[26] Madaghiele M., Demitri C., Sannino A., Ambrosio L. (2014). Polymeric hydrogels for burn wound care: Advanced skin wound dressings and regenerative templates. Burns Trauma.

[27] Nalampang K., Panjakha R., Molloy R., Tighe B.J. (2013). Structural effects in photopolymerized sodium AMPS hydrogels crosslinked with poly(ethylene glycol) diacrylate for use as burn dressings. J. Biomater. Sci. Polym. Ed..

[28] Edwards J. (2010). Hydrogels and their potential uses in burn wound management. Br. J. Nurs..

[29] Huang W., Wang Y., Huang Z., Wang X., Chen L., Zhang Y., Zhang L. (2018). On-Demand Dissolvable Self-Healing Hydrogel Based on Carboxymethyl Chitosan and Cellulose Nanocrystal for Deep Partial Thickness Burn Wound Healing. ACS Appl. Mater. Interfaces.

[30] Bankoti K., Rameshbabu A.P., Datta S., Goswami P., Roy M., Das D., Ghosh S.K., Das A.K., Mitra A., Pal S. (2021). Dual Functionalized Injectable Hybrid Extracellular Matrix Hydrogel for Burn Wounds. Biomacromolecules.

[31] Liang Y., He J., Guo B. (2021). Functional Hydrogels as Wound Dressing to Enhance Wound Healing. ACS Nano.

[32] Handley J.M. (2006). Adverse events associated with nonablative cutaneous visible and infrared laser treatment. J. Am. Acad. Dermatol..

[33] Cassuto D., Molla J.-F., Scrimali L., Sirago P. (2009). Right-left comparison study of hydrogel pad versus transparent fluid gel in patients with dermo-cosmetic lesions undergoing non-ablative laser therapy. J. Cosmet. Laser Ther..

[34] Stoica A.E., Chircov C., Grumezescu A.M. (2020). Hydrogel Dressings for the Treatment of Burn Wounds: An Up-To-Date Overview. Materials.

[35] Yao Y., Zhang A., Yuan C., Chen X., Liu Y. (2021). Recent trends on burn wound care: Hydrogel dressings and scaffolds. Biomater. Sci..

[36] Burd A. (2007). Evaluating the use of hydrogel sheet dressings in comprehensive burn wound care. Ostomy Wound Manag..

[37] Shu W., Wang Y., Zhang X., Li C., Le H., Chang F. (2021). Functional Hydrogel Dressings for Treatment of Burn Wounds. Front. Bioeng. Biotechnol..

[38] Khodja A.N., Mahlous M., Tahtat D., Benamer S., Youcef S.L., Chader H., Mouhoub L., Sedgelmaci M., Ammi N., Mansouri M.B. (2013). Evaluation of healing activity of PVA/chitosan hydrogels on deep second degree burn: Pharmacological and toxicological tests. Burns.

[39] Gharibi R., Shaker A., Rezapour-Lactoee A., Agarwal S. (2021). Antibacterial and Biocompatible Hydrogel Dressing Based on Gelatin- and Castor-Oil-Derived Biocidal Agent. ACS Biomater. Sci. Eng..

[40] Boonkaew B., Kempf M., Kimble R., Supaphol P., Cuttle L. (2014). Antimicrobial efficacy of a novel silver hydrogel dressing compared to two common silver burn wound dressings: Acticoat™ and PolyMem Silver^®^. Burns.

[41] Biazar E., Roveimiab Z., Shahhosseini G., Khataminezhad M., Zafari M., Majdi A. (2011). Biocompatibility Evaluation of a New Hydrogel Dressing Based on Polyvinylpyrrolidone/Polyethylene Glycol. J. Biomed. Biotechnol..

[42] Oliveira R.N., Rouzé R., Quilty B., Alves G., Soares G.D.A., Thiré R., McGuinness G. (2014). Mechanical properties and in vitro characterization of polyvinyl alcohol-nano-silver hydrogel wound dressings. Interface Focus.

[43] Kumar P.T.S., Lakshmanan V.K., Anilkumar T., Ramya C., Reshmi P., Unnikrishnan A., Nair S.V., Jayakumar R. (2012). Flexible and Microporous Chitosan Hydrogel/Nano ZnO Composite Bandages for Wound Dressing: In Vitro and In Vivo Evaluation. ACS Appl. Mater. Interfaces.

[44] Chakavala S., Patel N., Pate N.V., Thakkar V., Patel K., Gandhi T. (2012). Development and in vivo evaluation of silver sulfadiazine loaded hydrogel consisting polyvinyl alcohol and chitosan for severe burns. J. Pharm. Bioallied Sci..

[45] Zohdi R.M., Zakaria Z.A.B., Yusof N., Mustapha N.M., Abdullah M.N. (2012). Gelam (*Melaleuca* spp.) honey-based hydrogel as burn wound dressing. Evid. Based Complementary Altern. Med..

[46] Zhou G., Ruhan A., Ge H., Wang L., Liu M., Wang B., Su H., Yan M., Xi Y., Fan Y. (2014). Research on a novel poly (vinyl alcohol)/lysine/vanillin wound dressing: Biocompatibility, bioactivity and antimicrobial activity. Burns.

[47] Grolman J.M., Singh M., Mooney D., Eriksson E., Nuutila K. (2019). Antibiotic-Containing Agarose Hydrogel for Wound and Burn Care. J. Burn Care Res..

[48] Wang H., Liu Y., Cai K., Zhang B., Tang S., Zhang W., Liu W. (2021). Antibacterial polysaccharide-based hydrogel dressing containing plant essential oil for burn wound healing. Burn. Trauma.

[49] Zhu C., Zhao J., Kempe K., Wilson P., Wang J., Velkov T., Li J., Davis T., Whittaker M., Haddleton D.M. (2017). A Hydrogel-Based Localized Release of Colistin for Antimicrobial Treatment of Burn Wound Infection. Macromol. Biosci..

[50] Sun G., Zhang X., Shen Y.-I., Sebastian R., Dickinson L.E., Fox-Talbot K., Reinblatt M., Steenbergen C., Harmon J.W., Gerecht S. (2011). Dextran hydrogel scaffolds enhance angiogenic responses and promote complete skin regeneration during burn wound healing. Proc. Natl. Acad. Sci. USA.

[51] Wang P., Huang S., Hu Z., Yang W., Lan Y., Zhu J., Hancharou A., Guo R., Tang B. (2019). In situ formed anti-inflammatory hydrogel loading plasmid DNA encoding VEGF for burn wound healing. Acta Biomater..

[52] Natesan S., Zamora D.O., Wrice N.L., Baer D.G., Christy R.J. (2013). Bilayer Hydrogel with Autologous Stem Cells Derived from Debrided Human Burn Skin for Improved Skin Regeneration. J. Burn Care Res..

[53] Zhao X., Wu H., Guo B., Dong R., Qiu Y., Ma P.X. (2017). Antibacterial anti-oxidant electroactive injectable hydrogel as self-healing wound dressing with hemostasis and adhesiveness for cutaneous wound healing. Biomaterials.

[54] White-Dzuro C.G., Burns B., Pollins A., Rector J.A., Assi P.E., Thomas H.C., Jackson K., Perdikis G., Al Kassis S., Bellan L.M. (2021). Successful prevention of secondary burn progression using infliximab hydrogel: A murine model. Burns.

[55] Chakrabarti S., Mazumder B., Rajkonwar J., Pathak M.P., Patowary P., Chattopadhyay P. (2021). bFGF and collagen matrix hydrogel attenuates burn wound inflammation through activation of ERK and TRK pathway. Sci. Rep..

[56] Church D., Elsayed S., Reid O., Winston B., Lindsay R. (2006). Burn wound infections. Clin. Microbiol. Rev..

[57] Gomez R., Murray C.K., Hospenthal D.R., Cancio L.C., Renz E.M., Holcomb J.B., Wade C.E., Wolf S. (2009). Causes of Mortality by Autopsy Findings of Combat Casualties and Civilian Patients Admitted to a Burn Unit. J. Am. Coll. Surg..

[58] Altoparlak U., Erol S., Akcay M.N., Celebi F., Kadanali A. (2004). The time-related changes of antimicrobial resistance patterns and predominant bacterial profiles of burn wounds and body flora of burned patients. Burns.

[59] Kopecki Z. (2021). Development of next-generation antimicrobial hydrogel dressing to combat burn wound infection. Biosci. Rep..

[60] Drury J.L., Mooney D.J. (2003). Hydrogels for tissue engineering: Scaffold design variables and applications. Biomaterials.

[61] Konieczynska M.D., Villa-Camacho J.C., Ghobril C., Perez-Viloria M., Tevis K.M., Blessing W.A., Nazarian A., Rodriguez E.K., Grinstaff M.W. (2016). On-Demand Dissolution of a Dendritic Hydrogel-based Dressing for Second-Degree Burn Wounds through Thiol–Thioester Exchange Reaction. Angew. Chem..

[62] Konop M., Damps T., Misicka A., Rudnicka L. (2016). Certain Aspects of Silver and Silver Nanoparticles in Wound Care: A Minireview. J. Nanomater..

[63] Boonkaew B., Barber P.M., Rengpipat S., Supaphol P., Kempf M., He J., John V.T., Cuttle L. (2014). Development and Characterization of a Novel, Antimicrobial, Sterile Hydrogel Dressing for Burn Wounds: Single-Step Production with Gamma Irradiation Creates Silver Nanoparticles and Radical Polymerization. J. Pharm. Sci..

[64] Jodar K.S.P., Balca V.M., Chaud O.M.V., Tubino M., Yoshida V.M.H., Oliveira J.M., Vila M.M.D.C. (2015). Development and Characterization of a Hydrogel Containing Silver Sulfadiazine for Antimicrobial Topical Applications. J. Pharm. Sci..

[65] Chen X., Zhang H., Yang X., Zhang W., Jiang M., Wen T., Wang J., Guo R., Liu H. (2021). Preparation and Application of Quaternized Chitosan- and AgNPs-Base Synergistic Antibacterial Hydrogel for Burn Wound Healing. Molecules.

[66] Shen Y.-I., Song H.-H.G., Papa A., Burke J.A., Volk S.W., Gerecht S. (2015). Acellular Hydrogels for Regenerative Burn Wound Healing: Translation from a Porcine Model. J. Investig. Dermatol..

[67] Strużyna J., Pojda Z. (2015). Zastosowania komórek macierzystych z tkanki tłuszczowej w medycynie regeneracyjnej. Chir. Plast. Oparzenia Plast. Surg. Burn..

[68] Shukla L., Morrison W.A., Shayan R. (2015). Adipose-derived stem cells in radiotherapy injury: A new frontier. Front Surg..

[69] Suh A., Pham A., Cress M.J., Pincelli T., TerKonda S.P., Bruce A.J., Zubair A.C., Wolfram J., Shapiro S.A. (2019). Adipose-derived cellular and cell-derived regenerative therapies in dermatology and aesthetic rejuvenation. Ageing Res. Rev..

[70] Gimble J.M., Katz A.J., Bunnell B. (2007). Adipose-Derived Stem Cells for Regenerative Medicine. Circ. Res..

[71] Chen A., Zhang L., Chen P., Zhang C., Tang S., Chen X. (2021). Comparison of the Efficacy and Safety of Cell-Assisted Lipotransfer and Platelet-Rich Plasma Assisted Lipotransfer: What Should We Expect from a Systematic Review with Meta-Analysis?. Cell Transplant..

[72] Xiong S., Yi C., Pu L.L. (2020). An Overview of Principles and New Techniques for Facial Fat Grafting. Clin. Plast. Surg..

[73] O’Connor N.A., Syed A., Wong M., Hicks J., Nunez G., Jitianu A., Siler Z., Peterson M. (2020). Polydopamine Antioxidant Hydrogels for Wound Healing Applications. Gels.

[74] Ou Q., Zhang S., Fu C., Yu L., Xin P., Gu Z., Cao Z., Wu J., Wang Y. (2021). More natural more better: Triple natural anti-oxidant puerarin/ferulic acid/polydopamine incorporated hydrogel for wound healing. J. Nanobiotechnol..

[75] Zhang S., Ou Q., Xin P., Yuan Q., Wang Y., Wu J. (2019). Polydopamine/puerarin nanoparticle-incorporated hybrid hydrogels for enhanced wound healing. Biomater. Sci..

[76] Liang Y., Zhao X., Hu T., Han Y., Guo B. (2019). Mussel-inspired, antibacterial, conductive, antioxidant, injectable composite hydrogel wound dressing to promote the regeneration of infected skin. J. Colloid Interface Sci..

[77] Yuan Y., Shen S., Fan D. (2021). A physicochemical double cross-linked multifunctional hydrogel for dynamic burn wound healing: Shape adaptability, injectable self-healing property and enhanced adhesion. Biomaterials.

[78] Pan H., Fan D., Duan Z., Zhu C., Fu R., Li X. (2019). Non-stick hemostasis hydrogels as dressings with bacterial barrier activity for cutaneous wound healing. Mater. Sci. Eng. C.

[79] Zhang K., Xue K., Loh X. (2021). Thermo-Responsive Hydrogels: From Recent Progress to Biomedical Applications. Gels.

[80] Lei Z., Singh G., Min Z., Shixuan C., Xu K., Pengcheng X., Xueer W., Yinghua C., Lu Z., Lin Z. (2018). Bone marrow-derived mesenchymal stem cells laden novel thermo-sensitive hydrogel for the management of severe skin wound healing. Mater. Sci. Eng. C.

[81] Cao D., Chen X., Cao F., Guo W., Tang J., Cai C., Cui S., Yang X., Yu L., Su Y. (2021). An Intelligent Transdermal Formulation of ALA-Loaded Copolymer Thermogel with Spontaneous Asymmetry by Using Temperature-Induced Sol–Gel Transition and Gel–Sol (Suspension) Transition on Different Sides. Adv. Funct. Mater..

[82] Wang W., Wat E., Hui P.C., Chan B., Ng F.S., Kan C.W., Wang X., Hu H., Wong E.C., Lau C.B. (2016). Dual-functional transdermal drug delivery system with controllable drug loading based on thermosensitive poloxamer hydrogel for atopic dermatitis treatment. Sci. Rep..

[83] Djekic L., Krajisnik D., Martinovic M., Djordjevic D., Primorac M. (2015). Characterization of gelation proces and drug release profile of thermosensitive liquid lecithin/poloxamer 407 based gels as carriers for percutaneous delivery of ibuprofen. Int. J. Pharmaceut..

[84] Kim Y.C., Shin M.D., Hackett S.F., Hsueh H.T., Silva R.L., Date A., Han H., Kim B.-J., Xiao A., Kim Y. (2020). Gelling hypotonic polymer solution for extended topical drug delivery to the eye. Nat. Biomed. Eng..

[85] Zhu M., Wang J., Li N. (2018). A novel thermo-sensitive hydrogel-based on poly(N-isopropylacrylamide)/hyaluronic acid of ketoconazole for ophthalmic delivery. Artif. Cells Nanomed. Biotechnol..

[86] Tan G., Yu S., Li J., Pan W. (2017). Development and characterization of nanostructured lipid carriers based chitosan thermosensitive hydrogel for delivery of dexamethasone. Int. J. Biol. Macromol..

[87] Chu K., Chen L., Xu W., Li H., Zhang Y., Xie W., Zheng J. (2013). Preparation of a Paeonol-Containing Temperature-Sensitive In Situ Gel and Its Preliminary Efficacy on Allergic Rhinitis. Int. J. Mol. Sci..

[88] Crowe T., Greenlee M.H.W., Kanthasamy A., Hsu W.H. (2018). Mechanism of intranasal drug delivery directly to the brain. Life Sci..

[89] Abouhussein D.M., Khattab A., Bayoumi N.A., Mahmoud A.F., Sakr T.M. (2018). Brain targeted rivastigmine mucoadhesive thermosensitive in situ gel: Optimization, in vitro evaluation, radiolabeling, in vivo pharmacokinetics and biodistribution. J. Drug Deliv. Sci. Technol..

[90] Morelli L., Cappelluti M.A., Ricotti L., Lenardi C., Gerges I. (2017). An Injectable System for Local and Sustained Release of Antimicrobial Agents in the Periodontal Pocket. Macromol. Biosci..

[91] Zeng N., Seguin J., Destruel P.-L., Dumortier G., Maury M., Dhotel H., Bessodes M., Scherman D., Mignet N., Boudy V. (2017). Cyanine derivative as a suitable marker for thermosensitive in situ gelling delivery systems: In vitro and in vivo validation of a sustained buccal drug delivery. Int. J. Pharm..

[92] Liu M., Song X., Wen Y., Zhu J.L., Li J. (2017). Injectable Thermoresponsive Hydrogel Formed by Alginate-g-Poly (Nisopropylacrylamide) That Releases Doxorubicin-Encapsulated Micelles as a Smart Drug Delivery System. ACS Appl. Mater. Interfaces.

[93] Wu Y.-L., Wang H., Qiu Y.-K., Liow S.S., Li Z., Loh X.J. (2016). PHB-Based Gels as Delivery Agents of Chemotherapeutics for the Effective Shrinkage of Tumors. Adv. Heal. Mater..

[94] Jiang Y., Meng X., Wu Z., Qi X. (2016). Modified chitosan thermosensitive hydrogel enables sustained and efficient anti-tumor therapy via intratumoral injection. Carbohydr. Polym..

[95] Hang J., Zhang M., Lin R., Yun S., Du Y., Wang L., Yao Q., Zannettino A., Zhang H. (2020). Allogeneic primary mesenchymal stem/stromal cell aggregates within poly (N-isopropylacrylamide-co-acrylic acid) hydrogel for osteochondral regeneration. Appl. Mater. Today.

[96] Zhang Y., Zhang J., Chang F., Xu W., Ding J. (2018). Repair of full-thickness articular cartilage defect using stem cell-encapsulated thermogel. Mater. Sci. Eng. C.

[97] Liu H., Cheng Y., Chen J., Chang F., Wang J., Ding J., Chen X. (2018). Component effect of stem cell-loaded thermosensitive polypeptide hydrogels on cartilage repair. Acta Biomater..

[98] Dong L., Wang S.J., Zhao X.R., Zhu Y.F., Yu J.K. (2017). 3D-Printed Poly(epsilon-caprolactone) Scaffold Integrated with Cell-laden Chitosan Hydrogels for Bone Tissue Engineering. Sci. Rep..

[99] Kim M.H., Park H., Park W.H. (2018). Effect of pH and precursor salts on in situ formation of calcium phosphate nanoparticles in methylcellulose hydrogel. Carbohydr. Polym..

[100] Li R., Li Y., Wu Y., Zhao Y., Chen H., Yuan Y., Xu K., Zhang H., Lu Y., Wang J. (2018). Heparin-Poloxamer Thermosensitive Hydrogel Loaded with bFGF and NGF Enhances Peripheral Nerve Regeneration in Diabetic Rats. Biomaterials.

[101] Yu Y., Cheng Y., Tong J., Zhang L., Wei Y., Tian M. (2021). Recent advances in thermo-sensitive hydrogels for drug delivery. J. Mater. Chem. B.

[102] Huang H., Qi X., Chen Y., Wu Z. (2019). Thermo-sensitive hydrogels for delivering biotherapeutic molecules: A review. Saudi Pharm. J..

[103] Zhao Y.-Z., Jiang X., Lin Q., Xu H.-L., Huang Y.-D., Lu C.-T., Cai J. (2017). Thermosensitive heparin-poloxamer hydrogels enhance the effects of GDNF on neuronal circuit remodeling and neuroprotection after spinal cord injury. J. Biomed. Mater. Res. Part A.

[104] Goertz O., Abels C., Knie U., May T., Hirsch T., Daigeler A., Steinau H.-U., Langer S. (2010). Clinical Safety and Efficacy of a Novel Thermoreversible Polyhexanide-Preserved Wound Covering Gel. Eur. Surg. Res..

[105] Kramer A., Dissemond J., Kim S., Willy C., Mayer D., Papke R., Tuchmann F., Assadian O. (2018). Consensus on Wound Antisepsis: Update 2018. Ski. Pharmacol. Physiol..

[106] Jin J., Chen Z., Xiang Y., Tang T., Zhou H., Hong X., Fan H., Zhang X., Luo P., Ma B. (2020). Development of a PHMB hydrogel-modified wound scaffold dressing with antibacterial activity. Wound Repair Regen..

[107] Hirche C., Almeland S.K., Dheansa B., Fuchs P., Governa M., Hoeksema H., Korzeniowski T., Lumenta D.B., Marinescu S., Martinez-Mendez J.R. (2020). Eschar removal by bromelain based enzymatic debridement (Nexobrid^®^) in burns: European consensus guidelines update. Burns.

[108] Dhaliwal K., Lopez N. (2018). Hydrogel dressings and their application in burn wound care. Br. J. Community Nurs..

[109] Ou K.-L., Tzeng Y.-S., Chiao H.-Y., Chiu H.-T., Chen C.-Y., Chu T.-S., Huang D.-W., Hsu K.-F., Chang C.-K., Wang C.-H. (2021). Clinical Performance of Hydrogel-based Dressing in Facial Burn Wounds. Ann. Plast. Surg..

[110] Burks R.I. (2000). Ultrasound in wound care. Phys. Ther..

[111] Klucinec B., Scheidler M., Denegar C., Domholdt E., Burgess S. (2000). Effectiveness of Wound Care Products in the Transmission of Acoustic Energy. Phys. Ther..

