# Correction: Surowiecka et al. Hydrogels in Burn Wound Management—A Review. *Gels* 2022, *8*, 122

**DOI:** 10.3390/gels9010037

**Published:** 2022-12-31

**Authors:** Agnieszka Surowiecka, Jerzy Strużyna, Aleksandra Winiarska, Tomasz Korzeniowski

**Affiliations:** 1 East Center of Burns Treatment and Reconstructive Surgery, 21-010 Leczna, Poland; 2 Department of Plastic Surgery, Reconstructive Surgery and Burn Treatment, Medical University of Lublin, 21-093 Lublin, Poland; 3 Chair and Department of Didactics and Medical Simulation, Medical University of Lublin, 20-093 Lublin, Poland

In the original publication, there was a mistake in Table 2 as published [1]. The authors wish to make the following correction to their paper in relation to Reference 22 [2]. The citation was used incorrectly in Row 2, Columns 3 and 4 in Table 2, Page 9 [1]. 

The investigation by Holbert et al. aimed to examine the effectiveness of a hydrogel burn dressing as an analgesic adjunct to burn first aid in acute paediatric burn patients. No significant between-group differences in pain scores were found between paediatric burn patients who received hydrogel dressings and those who received standard care (2). A clear benefit of hydrogel dressings as an analgesic adjunct to burn first aid was not identified in the investigation (2). The corrected Table 2 appears below.

The authors state that the scientific conclusions are unaffected. This correction was approved by the Academic Editor. The original publication has also been updated.

## Figures and Tables

**Table 2:** Clinical evidence for hydrogel application.

Study	Study Type	Patients and Methods	Outcomes
Pre-hospital management of burns by the UK fire service [20]	A questionary	62 UK fire and rescue services were questioned about first aid in burns	76% use hydrogel dressing, while 37% would cool the wound with hydrogel
Effectiveness of a hydrogel dressing as an analgesic adjunct to first aid for the treatment of acute paediatric burn injuries: a prospective randomised controlled trial [22]	A prospective randomised controlled trial	Children were enrolled into two groups: intervention with inert hydrogel or control with polyvinylchloride film	No significant between-group differences in pain scores were found between 17 paediatric burn patients who received hydrogel dressings and those who received standard care
Evaluating the use of hydrogel sheet dressings in comprehensive burn wound care [36]	A prospective clinical observation	50 burn wounds in 30 patients treated with hydrogel sheets. Full-thickness and partial-thickness burn wounds, as well as the donor areas were treated.	No adverse events were reported. The hydrogel dressing reduced pain, improved wound healing
Clinical safety and efficacy of a novel thermoreversible polyhexanide-preserved wound covering gel [104]	A randomized controlled single-center study	44 patients, test group—hydrogel with polyhexanide, control group—ointment with sulfadiazine	There was less pain and wound staining in the test group. Hydrogels were safe and effective.
Clinical Performance of Hydrogel-based Dressing in Facial Burn Wounds: A Retrospective Observational Study [109]	A retrospective observational study	21 patients with burn enrolled in the study, a hydrogel mask was used. Full epithelialization took 10.86 days	Hydrogel mask improved healing and reduced scarring in a group of patients with second-degree facial burns.
